# Supplementary material for: Trends and associations of pulmonary nodule detection rates in China, 2019–2023: A multicenter cross-sectional study based on Real-World Data
Source: PLoS One. 2026 Feb 20;21(2):e0343207. doi: 10.1371/journal.pone.0343207 (PMC12923060; doi:10.1371/journal.pone.0343207)
Supplement: S1 Table — (DOCX) [file pone.0343207.s001.docx]

**Table S1** Impact of AI-Assisted Image Interpretation on Pulmonary Nodule Detection Rates (%) in Outpatient Populations

|  | **Outpatient males** | | | | **Outpatient females** | | | |
| --- | --- | --- | --- | --- | --- | --- | --- | --- |
|  | Pre- AI | Post-AI | χ^2^ | *P* | Pre- AI | Post-AI | χ^2^ | *P* |
| **AI-assisted image interpretation implementation prior to the initial emergence of COVID-19 (pre-2019)** | | | | | | | | |
| Hospital A | 14.82  (1109/7483) | 22.47  (1886/8393) | 151.289 | ＜0.001 | 18.08  (1111/6145) | 27.10  (1873/6911) | 150.163 | ＜0.001 |
| Hospital B | 19.68  (430/2195) | 25.52  (746/2923) | 24.923 | ＜0.001 | 27.26  (392/1438) | 31.15  (581/1865) | 5.921 | 0.015 |
| Hospital C | NA^a^ | 34.88  (1444/4140) | NA | NA | NA^a^ | 36.54  (802/2195) | NA | NA |
| **AI-assisted image interpretation implementation post to the cessation of COVID-19 emergency (post-December 2023)** | | | | | | | | |
| Hospital D | 14.06  (636/4524) | NA^b^ | NA | NA | 16.65  (609/3658) | NA^b^ | NA | NA |
| Hospital E | 39.84  (1595/4003) | NA^b^ | NA | NA | 40.32  (2155/5395) | NA^b^ | NA | NA |
| **AI-assisted image interpretation implementation prior to the initiation of COVID-19 vaccination (pre-December 2020)** | | | | | | | | |
| Hospital A | 14.82  (1109/7483) | 22.47  (1886/8393) | 151.289 | ＜0.001 | 18.08  (1111/6145) | 27.10  (1873/6911) | 150.163 | ＜0.001 |
| Hospital F | 12.82  (376/2932) | 15.81  (855/5408) | 13.473 | ＜0.001 | 13.87  (303/2185) | 18.39  (722/3926) | 20.571 | ＜0.001 |
| Hospital G | 29.10  (817/2808) | 21.59  (1587/7351) | 63.381 | ＜0.001 | 33.74  (588/1743) | 32.85  (1234/3756) | 0.417 | 0.518 |
| Hospital H | 14.59  (1366/9361) | 22.40  (2776/12397) | 210.541 | ＜0.001 | 15.30  (1342/8772) | 23.14  (2508/10837) | 189.050 | ＜0.001 |
| Hospital I | 71.48  (406/568) | 8.08  (516/6386) | 1822.974 | ＜0.001 | 44.64  (407/912) | 5.47  (338/6177) | 1295.444 | ＜0.001 |
| Hospital B | 19.68  (430/2195) | 25.52  (746/2923) | 24.923 | ＜0.001 | 27.26  (392/1438) | 31.15  (581/1865) | 5.921 | 0.015 |
| Hospital J | 50.86  (1510/2969) | 52.61  (1725/3279) | 1.908 | 0.167 | 55.83  (1488/2665) | 57.75  (1799/3115) | 2.154 | 0.142 |
| Hospital C | NA^a^ | 34.88  (1444/4140) | NA | NA | NA^a^ | 36.54  (802/2195) | NA | NA |

All hospital names in the table have been anonymized to comply with ethical requirements.

^a^Hospital C’s AI system was implemented earlier than the research data inclusion period, with its pre-AI data excluded from the study’s analytical framework.

^b^Hospital D and E’s AI system were implemented later than the research data inclusion period, with their post-AI data excluded from the study’s analytical framework.
