# Supplementary material for: Trends and associations of pulmonary nodule detection rates in China, 2019–2023: A multicenter cross-sectional study based on Real-World Data
Source: PLoS One. 2026 Feb 20;21(2):e0343207. doi: 10.1371/journal.pone.0343207 (PMC12923060; doi:10.1371/journal.pone.0343207)
Supplement: S2 Table — (DOCX) [file pone.0343207.s002.docx]

**Table S2** Impact of AI-Assisted Image Interpretation on Pulmonary Nodule Detection Rates (%) in Health Examination Populations

|  | **Health Examination Males** | | | | **Health Examination Females** | | | |
| --- | --- | --- | --- | --- | --- | --- | --- | --- |
|  | Pre-AI | Post-AI | χ^2^ | *P* | Pre-AI | Post-AI | χ^2^ | *P* |
| **AI-assisted image interpretation implementation prior to the initial emergence of COVID-19 (pre-2019)** | | | | | | | | |
| Hospital A | 9.42  (279/2962) | 17.30  (1153/6664) | 100.616 | ＜0.001 | 9.79  (145/1481) | 17.81  (668/3751) | 52.009 | ＜0.001 |
| Hospital B | 11.39  (539/4733) | 16.14  (716/4436) | 43.779 | ＜0.001 | 14.13  (241/1706) | 18.58  (340/1830) | 12.748 | ＜0.001 |
| Hospital C | NA^a^ | 47.37  (153/323) | NA | NA | NA^a^ | 41.51  (88/212) | NA | NA |
| **AI-assisted image interpretation implementation post to the cessation of COVID-19 emergency (post-December 2023)** | | | | | | | | |
| Hospital D | 17.16  (93/542) | NA^b^ | NA | NA | 17.77  (59/332) | NA^b^ | NA | NA |
| Hospital E | 73.08  (1925/2634) | NA^b^ | NA | NA | 77.07  (2122/2753) | NA^b^ | NA | NA |
| **AI-assisted image interpretation implementation prior to the initiation of COVID-19 vaccination (pre-December 2020)** | | | | | | | | |
| Hospital A | 9.42  (279/2962) | 17.30  (1153/6664) | 100.616 | ＜0.001 | 9.79  (145/1481) | 17.81  (668/3751) | 52.009 | ＜0.001 |
| Hospital F | 1.98  (28/1411) | 17.79  (450/2530) | 212.230 | ＜0.001 | 1.78  (10/561) | 24.90  (186/747) | 134.399 | ＜0.001 |
| Hospital G | 19.54  (186/952) | 33.55  (252/751) | 43.178 | ＜0.001 | 46.89  (151/322) | 27.42  (215/784) | 39.083 | ＜0.001 |
| Hospital H | 32.13  (401/1248) | 49.05  (927/1890) | 88.117 | ＜0.001 | 34.25  (112/327) | 47.79  (270/565) | 15.501 | ＜0.001 |
| Hospital I | 42.85  (329/768) | 0.40  (7/1746) | 829.613 | ＜0.001 | 68.73  (358/521) | 0.11  (2/1805) | 1454.546 | ＜0.001 |
| Hospital B | 11.39  (539/4733) | 16.14  (716/4436) | 43.779 | ＜0.001 | 14.13  (241/1706) | 18.58  (340/1830) | 12.748 | ＜0.001 |
| Hospital J | 0.00  (0/18) | 36.36  (8/22) | NA | NA | 100.00  (7/7) | 0.00  (0/8) | NA | NA |
| Hospital C | NA^a^ | 47.37  (153/323) | NA | NA | NA^a^ | 41.51  (88/212) | NA | NA |

All hospital names in the table have been anonymized to comply with ethical requirements.

^a^Hospital C’s AI system was implemented earlier than the research data inclusion period, with its pre-AI data excluded from the study’s analytical framework.

^b^Hospital D and E’s AI system were implemented later than the research data inclusion period, with their post-AI data excluded from the study’s analytical framework.
