# Supplementary material for: Trends and associations of pulmonary nodule detection rates in China, 2019–2023: A multicenter cross-sectional study based on Real-World Data
Source: PLoS One. 2026 Feb 20;21(2):e0343207. doi: 10.1371/journal.pone.0343207 (PMC12923060; doi:10.1371/journal.pone.0343207)
Supplement: S3 Table — (DOCX) [file pone.0343207.s003.docx]

**Table S3** Temporal Trends in CT-Diagnosed Pleural Effusion, Pneumonia, Suspected Lung Tumors, Lung Cancer, and Viral Pneumonia Detection Rates Across Distinct Subgroups

|  |  | Years | | | | |  |  |  |  |
| --- | --- | --- | --- | --- | --- | --- | --- | --- | --- | --- |
|  |  | 2019 | 2020 | 2021 | 2022 | 2023 | Wald χ^2^ | *P* for trend | H | *P* |
| **Pleural Effusion** | Outpatient males |  |  |  |  |  |  |  | 16.371 | 0.001 |
|  | University-affiliated/Provincial general hospitals | 7.40 | 6.61 | 6.89 | 7.07 | 6.42 | 58.834 | ＜0.001 |  |  |
|  | Municipal general hospitals | 9.27 | 7.04 | 8.04 | 7.81 | 7.54 | 96.941 | ＜0.001 |  |  |
|  | County hospitals | 12.50 | 17.67 | 17.87 | 14.45 | 14.86 | 81.118 | ＜0.001 |  |  |
|  | Specialized hospitals | 9.56 | 11.19 | 12.49 | 13.55 | 15.47 | 224.336 | ＜0.001 |  |  |
|  | Outpatient females |  |  |  |  |  |  |  | 16.714 | 0.001 |
|  | University-affiliated/Provincial general hospitals | 5.84 | 5.35 | 5.77 | 6.21 | 5.28 | 76.645 | ＜0.001 |  |  |
|  | Municipal general hospitals | 8.15 | 7.17 | 7.98 | 6.53 | 6.30 | 103.198 | ＜0.001 |  |  |
|  | County hospitals | 14.59 | 19.42 | 20.08 | 16.16 | 15.39 | 62.812 | ＜0.001 |  |  |
|  | Specialized hospitals | 6.95 | 7.88 | 9.80 | 10.82 | 11.65 | 157.860 | ＜0.001 |  |  |
|  | Health Examination Males |  |  |  |  |  |  |  | NA | NA |
|  | University-affiliated/provincial general hospitals | 0.35 | 0.88 | 0.62 | 0.52 | 1.14 | 154.771 | ＜0.001 |  |  |
|  | Municipal general hospitals | 0.93 | 0.72 | 0.85 | 0.94 | 1.19 | 41.257 | ＜0.001 |  |  |
|  | County hospitals | 0.00 | 0.00 | 0.50 | 1.00 | 1.71 | 3.835 | 0.429 |  |  |
|  | Specialized hospitals | NA | NA | NA | 0.00 | NA | NA | NA |  |  |
|  | Health Examination Females |  |  |  |  |  |  |  | NA | NA |
|  | University-affiliated/provincial general hospitals | 0.60 | 0.95 | 0.73 | 0.75 | 1.34 | 93.435 | ＜0.001 |  |  |
|  | Municipal general hospitals | 1.50 | 1.10 | 1.10 | 1.41 | 1.74 | 49.385 | ＜0.001 |  |  |
|  | County hospitals | 0.00 | 0.00 | 0.34 | 0.31 | 1.16 | 1.628 | 0.804 |  |  |
|  | Specialized hospitals | NA | NA | NA | 0.00 | NA | NA | NA |  |  |
| **Pneumonia** | Outpatient males |  |  |  |  |  |  |  | 14.520 | 0.002 |
|  | University-affiliated/Provincial general hospitals | 11.99 | 12.39 | 11.96 | 12.90 | 8.81 | 851.595 | ＜0.001 |  |  |
|  | Municipal general hospitals | 13.30 | 11.70 | 11.82 | 13.60 | 13.62 | 122.435 | ＜0.001 |  |  |
|  | County hospitals | 21.33 | 20.48 | 24.71 | 25.79 | 23.68 | 59.485 | ＜0.001 |  |  |
|  | Specialized hospitals | 14.86 | 19.72 | 24.42 | 27.34 | 32.62 | 1219.767 | ＜0.001 |  |  |
|  | Outpatient females |  |  |  |  |  |  |  | 15.754 | 0.001 |
|  | University-affiliated/Provincial general hospitals | 11.88 | 11.19 | 10.97 | 12.09 | 7.89 | 1011.435 | ＜0.001 |  |  |
|  | Municipal general hospitals | 16.96 | 15.92 | 15.97 | 14.68 | 17.37 | 100.365 | ＜0.001 |  |  |
|  | County hospitals | 26.83 | 20.50 | 25.72 | 27.58 | 27.66 | 66.565 | ＜0.001 |  |  |
|  | Specialized hospitals | 17.22 | 21.87 | 27.76 | 30.19 | 34.65 | 841.349 | ＜0.001 |  |  |
|  | Health Examination Males |  |  |  |  |  |  |  | NA | NA |
|  | University-affiliated/provincial general hospitals | 2.62 | 2.61 | 4.45 | 4.27 | 2.83 | 291.901 | ＜0.001 |  |  |
|  | Municipal general hospitals | 3.78 | 6.65 | 7.38 | 8.71 | 14.91 | 2207.944 | ＜0.001 |  |  |
|  | County hospitals | 21.74 | 8.16 | 4.99 | 6.98 | 12.82 | 29.378 | ＜0.001 |  |  |
|  | Specialized hospitals | NA | NA | NA | 0.00 | NA | NA | NA |  |  |
|  | Health Examination Females |  |  |  |  |  |  |  | NA | NA |
|  | University-affiliated/provincial general hospitals | 2.90 | 2.68 | 4.12 | 3.07 | 1.93 | 238.805 | ＜0.001 |  |  |
|  | Municipal general hospitals | 3.21 | 6.43 | 7.54 | 9.84 | 13.44 | 1042.244 | ＜0.001 |  |  |
|  | County hospitals | 20.59 | 3.13 | 4.45 | 4.31 | 9.25 | 16.807 | 0.002 |  |  |
|  | Specialized hospitals | NA | NA | NA | 0.00 | NA | NA | NA |  |  |
| **Lung Tumor** | Outpatient males |  |  |  |  |  |  |  | 13.217 | 0.004 |
|  | University-affiliated/Provincial general hospitals | 4.08 | 3.52 | 3.62 | 3.25 | 2.77 | 189.326 | ＜0.001 |  |  |
|  | Municipal general hospitals | 3.63 | 3.16 | 3.25 | 2.83 | 2.67 | 91.347 | ＜0.001 |  |  |
|  | County hospitals | 3.34 | 5.41 | 4.81 | 5.11 | 3.70 | 37.686 | ＜0.001 |  |  |
|  | Specialized hospitals | 23.47 | 26.53 | 29.69 | 30.30 | 35.40 | 472.832 | ＜0.001 |  |  |
|  | Outpatient females |  |  |  |  |  |  |  | 15.274 | 0.002 |
|  | University-affiliated/Provincial general hospitals | 3.63 | 3.16 | 3.25 | 2.83 | 2.67 | 118.259 | ＜0.001 |  |  |
|  | Municipal general hospitals | 3.95 | 3.18 | 3.91 | 3.22 | 3.07 | 51.126 | ＜0.001 |  |  |
|  | County hospitals | 3.34 | 5.41 | 4.81 | 5.11 | 3.70 | 65.503 | ＜0.001 |  |  |
|  | Specialized hospitals | 25.82 | 29.89 | 34.60 | 34.77 | 37.10 | 321.902 | ＜0.001 |  |  |
|  | Health Examination Males |  |  |  |  |  |  |  | NA | NA |
|  | University-affiliated/provincial general hospitals | 0.64 | 0.61 | 0.69 | 0.63 | 0.48 | 16.093 | 0.003 |  |  |
|  | Municipal general hospitals | 1.39 | 1.09 | 0.95 | 0.44 | 1.13 | 138.277 | ＜0.001 |  |  |
|  | County hospitals | 4.35 | 2.04 | 0.83 | 0.57 | 1.20 | 4.351 | 0.361 |  |  |
|  | Specialized hospitals | NA | NA | NA | 0.09 | NA | NA | NA |  |  |
|  | Health Examination Females |  |  |  |  |  |  |  | NA | NA |
|  | University-affiliated/provincial general hospitals | 0.72 | 0.69 | 0.66 | 0.63 | 0.51 | 10.848 | 0.028 |  |  |
|  | Municipal general hospitals | 1.56 | 0.98 | 0.99 | 0.56 | 1.20 | 75.160 | ＜0.001 |  |  |
|  | County hospitals | 0.00 | 0.00 | 1.37 | 0.31 | 0.58 | 2.067 | 0.723 |  |  |
|  | Specialized hospitals | NA | NA | NA | 0.00 | NA | NA | NA |  |  |
| **Lung Cancer** | Outpatient males |  |  |  |  |  |  |  | 16.910 | 0.001 |
|  | University-affiliated/Provincial general hospitals | 5.51 | 5.12 | 5.94 | 5.81 | 5.09 | 80.771 | ＜0.001 |  |  |
|  | Municipal general hospitals | 2.66 | 1.63 | 2.52 | 2.44 | 2.66 | 124.531 | ＜0.001 |  |  |
|  | County hospitals | 2.92 | 3.64 | 3.73 | 3.03 | 2.22 | 24.540 | ＜0.001 |  |  |
|  | Specialized hospitals | 6.32 | 8.43 | 9.05 | 9.95 | 11.43 | 206.454 | ＜0.001 |  |  |
|  | Outpatient females |  |  |  |  |  |  |  | 17.331 | 0.001 |
|  | University-affiliated/Provincial general hospitals | 7.03 | 6.99 | 8.46 | 8.90 | 7.18 | 276.912 | ＜0.001 |  |  |
|  | Municipal general hospitals | 3.18 | 2.16 | 4.30 | 3.94 | 4.91 | 370.206 | ＜0.001 |  |  |
|  | County hospitals | 2.11 | 1.96 | 1.83 | 1.81 | 1.52 | 3.571 | 0.467 |  |  |
|  | Specialized hospitals | 8.07 | 10.48 | 11.91 | 12.09 | 13.44 | 142.003 | ＜0.001 |  |  |
|  | Health Examination Males |  |  |  |  |  |  |  | NA | NA |
|  | University-affiliated/provincial general hospitals | 0.50 | 0.59 | 0.67 | 0.64 | 0.66 | 6.588 | 0.159 |  |  |
|  | Municipal general hospitals | 0.18 | 0.15 | 0.14 | 0.26 | 0.31 | 34.744 | ＜0.001 |  |  |
|  | County hospitals | 0.00 | 0.00 | 0.00 | 0.28 | 0.34 | NA | NA |  |  |
|  | Specialized hospitals | NA | NA | NA | 0.00 | NA | NA | NA |  |  |
|  | Health Examination Females |  |  |  |  |  |  |  | NA | NA |
|  | University-affiliated/provincial general hospitals | 0.75 | 0.86 | 1.21 | 1.07 | 1.02 | 20.363 | ＜0.001 |  |  |
|  | Municipal general hospitals | 0.15 | 0.19 | 0.20 | 0.30 | 0.37 | 22.534 | ＜0.001 |  |  |
|  | County hospitals | 0.00 | 0.00 | 0.00 | 0.00 | 0.00 | NA | NA |  |  |
|  | Specialized hospitals | NA | NA | NA | 0.00 | NA | NA | NA |  |  |
| **Viral Pneumonia** | Outpatient males |  |  |  |  |  |  |  | 0.518 | 0.915 |
|  | University-affiliated/Provincial general hospitals | 0.07 | 0.18 | 0.04 | 0.61 | 0.28 | 385.819 | ＜0.001 |  |  |
|  | Municipal general hospitals | 0.01 | 0.16 | 0.18 | 2.85 | 0.54 | 1282.515 | ＜0.001 |  |  |
|  | County hospitals | 0.64 | 0.00 | 0.02 | 0.97 | 0.34 | 28.253 | ＜0.001 |  |  |
|  | Specialized hospitals | 0.00 | 0.05 | 0.07 | 0.65 | 0.26 | 79.662 | ＜0.001 |  |  |
|  | Outpatient females |  |  |  |  |  |  |  | 1.248 | 0.741 |
|  | University-affiliated/Provincial general hospitals | 0.02 | 0.16 | 0.02 | 0.57 | 0.23 | 353.852 | ＜0.001 |  |  |
|  | Municipal general hospitals | 0.00 | 0.15 | 0.23 | 2.89 | 0.49 | 1038.627 | ＜0.001 |  |  |
|  | County hospitals | 0.43 | 0.03 | 0.00 | 1.18 | 0.26 | 35.811 | ＜0.001 |  |  |
|  | Specialized hospitals | 0.00 | 0.01 | 0.08 | 0.60 | 0.14 | 57.186 | ＜0.001 |  |  |
|  | Health Examination Males |  |  |  |  |  |  |  | NA | NA |
|  | University-affiliated/provincial general hospitals | 0.01 | 0.00 | 0.00 | 0.07 | 0.04 | 15.768 | 0.003 |  |  |
|  | Municipal general hospitals | 0.01 | 0.00 | 0.00 | 0.07 | 0.10 | 19.369 | 0.001 |  |  |
|  | County hospitals | 0.00 | 0.00 | 0.00 | 0.00 | 0.17 | NA | NA |  |  |
|  | Specialized hospitals | NA | NA | NA | 0.00 | NA | NA | NA |  |  |
|  | Health Examination Females |  |  |  |  |  |  |  | NA | NA |
|  | University-affiliated/provincial general hospitals | 0.00 | 0.00 | 0.00 | 0.06 | 0.04 | 2.122 | 0.713 |  |  |
|  | Municipal general hospitals | 0.00 | 0.00 | 0.01 | 0.11 | 0.11 | 12.929 | 0.012 |  |  |
|  | County hospitals | 0.00 | 0.00 | 0.00 | 0.00 | 0.00 | NA | NA |  |  |
|  | Specialized hospitals | NA | NA | NA | 0.00 | NA | NA | NA |  |  |
