# Supplementary material for: Trends and associations of pulmonary nodule detection rates in China, 2019–2023: A multicenter cross-sectional study based on Real-World Data
Source: PLoS One. 2026 Feb 20;21(2):e0343207. doi: 10.1371/journal.pone.0343207 (PMC12923060; doi:10.1371/journal.pone.0343207)
Supplement: S4 Table — (Including Number of Cases/ Total Samples). (DOCX) [file pone.0343207.s004.docx]

**Table S4. Temporal Trends in Pulmonary Nodule Detection Rates (%) Across Distinct Clinical Populations and Gender Subgroups. (Including Number of Cases / Total Samples)**

|  | Years | | | | |  |  |  |  |
| --- | --- | --- | --- | --- | --- | --- | --- | --- | --- |
|  | 2019 | 2020 | 2021 | 2022 | 2023 | Waldχ^2^ | *P* for trend | U | *P* |
| Outpatient populations | 28.21  (41615/147542) | 27.21  (59940/220249) | 37.16  (78218/210488) | 40.21  (91614/227833) | 49.52  (110948/224054) | 29372.929 | ＜0.001 | 17.000 | 0.421 |
| Male | 27.31  (21902/80197) | 25.16  (31023/123285) | 34.21  (39047/114153) | 37.17  (43795/117822) | 46.81  (69031/147467) | 16275.107 | ＜0.001 |  |  |
| Female | 29.27  (19713/67345) | 29.82  (28917/96964) | 40.66  (39171/96335) | 43.47  (47819/110011) | 51.21  (76318/149026) | 15265.262 | ＜0.001 |  |  |
| Health Examination populations | 32.08  (16203/50514) | 33.46  (29211/87306) | 43.80  (47849/109236) | 44.19  (52165/118040) | 48.32  (72023/149049) | 7365.155 | ＜0.001 | 16.000 | 0.548 |
| Male | 29.83  (9542/31987) | 32.93  (17695/53737) | 43.27  (28013/64740) | 44.14  (30378/68819) | 47.47  (39752/83738) | 4867.719 | ＜0.001 |  |  |
| Female | 35.95  (6661/18527) | 34.31  (11516/33569) | 44.58  (19836/44496) | 44.26  (21787/49221) | 49.41  (32271/65311) | 2518.971 | ＜0.001 |  |  |
