# Supplementary material for: Trends and associations of pulmonary nodule detection rates in China, 2019–2023: A multicenter cross-sectional study based on Real-World Data
Source: PLoS One. 2026 Feb 20;21(2):e0343207. doi: 10.1371/journal.pone.0343207 (PMC12923060; doi:10.1371/journal.pone.0343207)
Supplement: S5 Table — (Including Number of Cases/ Total Samples). (DOCX) [file pone.0343207.s005.docx]

**Table S5. Temporal Trends in Pulmonary Nodule Detection Rates (%) Across Distinct Clinical Populations and Gender Subgroups after Adjusting for AI-related effects. (Including Number of Cases / Total Samples)**

|  | Years | | | | |  |  |
| --- | --- | --- | --- | --- | --- | --- | --- |
|  | 2019 | 2020 | 2021 | 2022 | 2023 | Waldχ^2^ | *P* for trend |
| **AI-assisted image interpretation implementation prior to the initial emergence of COVID-19 (pre-2019)** | | | | | | | |
| Outpatient populations | 31.81  (9847/30955) | 32.82  (16172/49271) | 49.22  (23686/48124) | 54.13  (29314/54153) | 55.47  (36426/65664) | 9696.036 | ＜0.001 |
| Male | 31.71  (5493/17321) | 29.21  (8500/29103) | 45.13  (11991/26571) | 51.00  (13907/27269) | 50.99  (16912/33167) | 4686.051 | ＜0.001 |
| Female | 31.93  (4354/13634) | 38.04  (7672/20168) | 54.26  (11695/21553) | 57.31  (15407/26884) | 60.05  (19514/32497) | 4771.218 | ＜0.001 |
| Health Examination populations | 30.10  (9141/30366) | 36.00  (15453/42929) | 45.05  (23343/51818) | 46.02  (22798/49539) | 44.61  (29600/66352) | 3063.885 | ＜0.001 |
| Male | 29.29  (5614/19165) | 34.90  (9420/26992) | 43.45  (13234/30459) | 44.47  (13631/30650) | 42.91  (16409/38237) | 1726.886 | ＜0.001 |
| Female | 31.49  (3527/11201) | 37.86  (6033/15937) | 47.33  (10109/21359) | 48.53  (9167/18889) | 46.92  (13191/28115) | 1286.656 | ＜0.001 |
| **AI-assisted image interpretation implementation post to the cessation of COVID-19 emergency (post-December 2023)** | | | | | | | |
| Outpatient populations | 13.07  (919/7031) | 13.09  (1864/14241) | 21.98  (3057/13908) | 26.71  (4178/15643) | 28.41  (4995/17580) | 1551.676 | ＜0.001 |
| Male | 12.85  (522/4062) | 12.46  (1027/8245) | 20.31  (1532/7542) | 24.86  (1992/8013) | 26.16  (2231/8527) | 717.997 | ＜0.001 |
| Female | 13.37  (397/2969) | 13.96  (837/5996) | 23.96  (1525/6366) | 28.65  (2186/7630) | 30.53  (2764/9053) | 781.659 | ＜0.001 |
| Health Examination populations | 61.32  (241/393) | 45.60  (554/1215) | 65.66  (1782/2714) | 63.96  (2328/3640) | 67.07  (4199/6261) | 200.377 | ＜0.001 |
| Male | 60.95  (167/274) | 41.73  (343/822) | 62.79  (1041/1658) | 63.34  (1398/2207) | 63.54  (2018/3176) | 137.911 | ＜0.001 |
| Female | 62.18  (74/119) | 53.69  (211/393) | 70.17  (741/1056) | 64.90  (930/1433) | 70.70  (2181/3085) | 56.568 | ＜0.001 |
| **AI-assisted image interpretation implementation prior to the initiation of COVID-19 vaccination (pre-December 2020)** | | | | | | | |
| Outpatient populations | 28.01  (18450/65870) | 27.02  (30232/111903) | 37.76  (40890/108301) | 39.64  (46751/117936) | 52.44  (93843/178947) | 23022.052 | ＜0.001 |
| Male | 27.72  (9968/35959) | 24.97  (15959/63924) | 35.18  (20757/58999) | 37.28  (22595/60607) | 50.94  (44842/88032) | 12356.769 | ＜0.001 |
| Female | 28.36  (8482/29911) | 29.75  (14273/47979) | 40.84  (20133/49302) | 42.14  (24165/57329) | 53.90  (49001/90915) | 10289.213 | ＜0.001 |
| Health Examination populations | 29.38  (10723/36501) | 33.04  (17770/53777) | 44.10  (28343/64264) | 43.55  (28874/66299) | 45.11  (43860/97238) | 4497.136 | ＜0.001 |
| Male | 27.83  (6558/23562) | 32.61  (11064/33931) | 43.17  (16453/38108) | 43.42  (17506/40319) | 44.78  (24340/54351) | 3054.310 | ＜0.001 |
| Female | 32.19  (4165/12939) | 33.79  (6707/19846) | 45.46  (11890/26156) | 43.76  (11368/25980) | 45.51  (19520/42887) | 1424.789 | ＜0.001 |
