# Supplementary material for: Trends and associations of pulmonary nodule detection rates in China, 2019–2023: A multicenter cross-sectional study based on Real-World Data
Source: PLoS One. 2026 Feb 20;21(2):e0343207. doi: 10.1371/journal.pone.0343207 (PMC12923060; doi:10.1371/journal.pone.0343207)
Supplement: S6 Table — (Including Number of Cases/ Total Samples). (DOCX) [file pone.0343207.s006.docx]

**Table S6. Temporal Trends in Pulmonary Nodule Detection Rates (%) Across Hospital Tier and Specialty Subgroups. (Including Number of Cases / Total Samples)**

|  | Years | | | | |  |  |  |  |
| --- | --- | --- | --- | --- | --- | --- | --- | --- | --- |
|  | 2019 | 2020 | 2021 | 2022 | 2023 | Wald χ^2^ | *P* for trend | H | *P/Adj. P* |
| Outpatient males |  |  |  |  |  |  |  | 8.737 | 0.033 ^a^ |
| University-affiliated/Provincial general hospitals | 31.27  (14191/45376) | 28.66  (17917/62522) | 36.96  (22595/61130) | 38.73  (25178/65009) | 50.54  (46725/92455) | 9077.117 | ＜0.001 |  | 0.033 ^b^ |
| Municipal general hospitals | 26.72  (4979/18632) | 21.92  (9475/43225) | 34.30  (11773/34320) | 37.99  (13517/35579) | 42.58  (15779/37059) | 4594.631 | ＜0.001 |  |  |
| County hospitals | 16.02  (778/4857) | 18.60  (939/5049) | 19.70  (1097/5569) | 22.21  (1282/5773) | 23.49  (1111/4730) | 107.272 | ＜0.001 |  | 0.033^b^ |
| Specialized hospitals | 17.24  (1954/11332) | 21.55  (2692/12489) | 27.27  (3582/13134) | 33.31  (3818/11461) | 40.96  (5416/13223) | 2083.69 | ＜0.001 |  |  |
| Outpatient females |  |  |  |  |  |  |  | 10.349 | 0.016 ^a^ |
| University-affiliated/Provincial general hospitals | 32.11  (13834/43086) | 32.37  (18366/56744) | 42.83  (24619/57481) | 46.20  (28714/62150) | 53.34  (53005/99365) | 9096.226 | ＜0.001 |  | 0.017 ^b^ |
| Municipal general hospitals | 28.04  (3727/13290) | 27.16  (7803/28734) | 41.43  (10590/25560) | 40.86  (13804/33786) | 48.91  (17106/34973) | 3843.258 | ＜0.001 |  |  |
| County hospitals | 13.98  (458/3276) | 18.94  (666/3517) | 19.16  (795/4149) | 23.03  (1016/4412) | 22.79  (782/3431) | 119.260 | ＜0.001 |  | 0.017 ^b^ |
| Specialized hospitals | 22.02  (1694/7693) | 26.13  (2082/7969) | 34.63  (3167/9145) | 44.34  (4285/9663) | 48.19  (5425/11257) | 1941.96 | ＜0.001 |  |  |
| Health Examination Males |  |  |  |  |  |  |  | 13.018 | 0.005 ^a^ |
| University-affiliated/provincial general hospitals | 33.00  (5737/17384) | 37.21  (9332/25082) | 48.66  (13913/28590) | 50.41  (16080/31898) | 54.68  (25415/46478) | 3547.930 | ＜0.001 |  |  |
| Municipal general hospitals | 26.07  (3801/14582) | 29.23  (8362/28606) | 39.40  (14008/35549) | 38.62  (13983/36208) | 38.51  (14122/36675) | 1563.607 | ＜0.001 |  |  |
| County hospitals | 26.09  (6/23) | 2.04  (1/49) | 15.31  (92/601) | 44·73  (314/702) | 36.75  (215/585) | 133.390 | ＜0.001 |  |  |
| Specialized hospitals | 0  (0/0) | 0  (0/0) | 0  (0/0) | 9.09  (1/11) | 0  (0/0) | NA | NA |  |  |
| Health Examination Females |  |  |  |  |  |  |  | 16.046 | 0.001 ^a^ |
| University-affiliated/provincial general hospitals | 39.23  (4558/11618) | 37.37  (6196/16582) | 48.55  (9779/20144) | 47.79  (12414/25976) | 53.53  (20767/38795) | 1561.044 | ＜0.001 |  |  |
| Municipal general hospitals | 30.50  (2097/6875) | 31.37  (5318/16955) | 41.63  (10017/24060) | 40.59  (9266/22831) | 43.47  (11451/26343) | 924.588 | ＜0.001 |  |  |
| County hospitals | 17.65  (6/34) | 6.25  (2/32) | 13.70  (40/292) | 30.46  (99/325) | 30.64  (53/173) | 32.855 | ＜0.001 |  |  |
| Specialized hospitals | 0  (0/0) | 0  (0/0) | 0  (0/0) | 8.99  (8/89) | 0  (0/0) | NA | NA |  |  |

^a^Kruskal-Wallis test *P*-value.

^b^Bonferroni-corrected Adjusted *P*-value.
