# Supplementary material for: Trends and associations of pulmonary nodule detection rates in China, 2019–2023: A multicenter cross-sectional study based on Real-World Data
Source: PLoS One. 2026 Feb 20;21(2):e0343207. doi: 10.1371/journal.pone.0343207 (PMC12923060; doi:10.1371/journal.pone.0343207)
Supplement: Attachment — Submitted filename: Reviewers report.pdf [file pone.0343207.s007.pdf]

I acknowledge the ambition and scale of this multicenter cross-sectional study. The topic of post-COVID-19 pulmonary sequelae and the impact of the pandemic on lung health are undoubtedly crucial. However, for this manuscript to achieve its envisioned position in the field, several fundamental epidemiological and methodological deficiencies need to be addressed. My critique focuses on areas where the study's design and interpretation fall short of robust epidemiological standards, particularly when drawing conclusions about "determinants" and causal links.

Major concerns:

1. **Limitations of Cross-Sectional Design for Causal Inference:**

    a. I am particularly concerned about the use of a strong word, "Determinants" in this study title! Of note, keenly considering **"Determinants" vs. "Associations",** here explicitly suggesting a causal relationship. However, a multicenter *cross-sectional* study, even with temporal trend analysis, is inherently limited in establishing causality. It can identify associations and trends, but cannot definitively prove that SARS-CoV-2 infection or vaccination *caused* or *prevented* specific changes in nodule *incidence*. The observed "surges" are co-temporal with pandemic events and AI adoption, but co-occurrence does not equal causation. For a "great" paper in epidemiology, the language must reflect the true strength of the evidence.

    b. Also, in terms of "Reverse Causality/Bias", could changes in health-seeking behavior, increased awareness due to the pandemic, or altered screening guidelines contribute to increased *detection* rates rather than an actual increase in nodule *prevalence* or *incidence*? This is a significant confounding factor that cross-sectional data struggles to disentangle.

2. **Lack of Individual-Level Data on Key Exposure and Confounding Variables:**

    a. The study correlates nodule detection trends with population-level "surges in population-level infection rates" and "pandemic peaks." However, there's no individual-level data on who was infected, when, or with what severity. Without this, linking *individual* nodule detection to *individual* infection history is impossible. Many observed nodules could be incidental, pre-existing, or related to

other factors putting the conclusion at the risk of misleading the public and particularly the policy makers.

b. Similarly, the conclusion that "COVID-19 vaccination did not accelerate detection but may have slowed it short-term" is based on population-level vaccination rollout periods. There's no data to show the vaccination status of individuals with detected nodules. This is a critical gap for making any claim about vaccine impact. Observational data at a population level is highly susceptible to ecological fallacy and unmeasured confounders.

c. There are several other confounders noted in the paper. For instance, for pulmonary nodules, factors like smoking history, age, occupation, environmental exposures, and pre-existing lung conditions are paramount. The study mentions "health exam populations" which might differ significantly from "outpatient populations" in these baseline characteristics. Without adjusting for these at the individual level, it's difficult to attribute observed trends solely to COVID-19-related factors or AI.

3. **"Detection Rate" vs. "Incidence/Prevalence":**

   a. I noticed that the manuscript consistently reported "**pulmonary nodule detection rates**." An increase in *detection* can be driven by improved technology (AI), increased screening volume, or greater scrutiny by radiologists, independent of a true increase in nodule *incidence*. The paper acknowledges AI's role as a confounder and attempts to adjust for it, which is good, but the overall increase in CT scans during the pandemic (due to respiratory symptoms) could also inflate detection. This distinction needs to be central to the interpretation, especially for epidemiological conclusions.

4. **Definition and Standardization of "Pulmonary Nodule":**

   a. The manuscript states nodules were "CT-diagnosed" and reviewed by AI and radiologists. However, specific criteria for what constitutes a "pulmonary nodule" (size, morphology, consistency) and the follow-up protocols might vary across 23 diverse centers (university-affiliated, municipal, county, specialized hospitals).

This potential lack of standardization could introduce heterogeneity and bias into the aggregated "detection rates." More information to accurately portray standardized term by definitive criteria is required.

5. **Generalizability and Representativeness of the Study Population:**

   a. The study analyzes data from "outpatient and health exam populations" from 23 centers. While substantial, these populations may not be representative of the general Chinese population. Outpatients are by definition seeking medical care, often due to symptoms, while health exam participants may be self-selected or screened for specific purposes. This limits the generalizability of the "detection rates" to the broader population and could risk erroneous outcome and mislead the public.

6. **Mechanistic Speculation Without Direct Evidence:**

   a. The discussion touches upon mechanisms like pulmonary fibrosis and microvascular damage as pathophysiological bases for Long COVID. While relevant to the broader field, the current study does not provide direct evidence linking these mechanisms to the *detected pulmonary nodules* in their study population. The findings are primarily statistical trends of detection, not pathological characterizations. The authors need to address this against overstretching the fact beyond their data.

7. **Regarding the Timing of Ethical Data Access:**

   a. **Lines 130-131:** The methods state: "The data were accessed for research purposes from 03/01/2025 to 26/03/2025." Given the server time is 2025-12-03, this indicates the data access period *has not yet occurred*. While possibly a typo (e.g., 2024), this raises a flag about the practical feasibility of the study timeframe and reflects a potential lack of precision in documentation. If it's indeed data accessed *in the future*, it undermines the credibility of the research as presented.

Minor Concerns:

1. Redundancy of "P for trend <0.001" was noted: While important, simply stating "<0.001" for every P-value makes it harder to discern relative significance or subtle differences if they exist. More precise P-values (e.g., 1.2e-5, 0.0003) would be more informative.
2. The table presents (number of cases / total samples). While clear, the overall sample sizes for each year/subgroup are very large. This level of detail is good, but ensuring readability with large numbers is key.
